# PPAR Alpha Regulation of the Immune Response and Autoimmune Encephalomyelitis

**DOI:** 10.1155/2008/546753

**Published:** 2008-07-14

**Authors:** Yuhong Yang, Anne R. Gocke, Amy Lovett-Racke, Paul D. Drew, Michael K. Racke

**Affiliations:** ^1^Department of Neurology, The Ohio State University Medical Center, 1654 Upham Drive, 445 Means Hall, Columbus, OH 43210, USA; ^2^Department of Neurology, University of Texas Southwestern Medical Center, Dallas, TX 75390, USA; ^3^Department of Molecular Virology, Immunology & Medical Genetics, The Ohio State University Medical Center, Columbus, OH 43210, USA; ^4^Department of Neurobiology and Developmental Sciences, University of Arkansas for Medical Sciences, Little Rock, AR 72205, USA

## Abstract

PPARs are members of the steroid hormone nuclear receptor superfamily and play an important role in the regulation of lipid metabolism, energy balance, artherosclerosis and glucose control. Recent studies suggest that they play an important role in regulating inflammation. This review will focus on PPAR-*α* regulation of the immune response. We describe how PPAR-*α* regulates differentiation of T cells by transactivation and/or interaction with other transcription factors. Moreover, PPAR-*α* agonists have been shown to ameliorate experimental autoimmune encephalomyelitis (EAE) in mice, suggesting that they could provide a therapy for human autoimmune diseases such as multiple sclerosis.

## 1. PEROXISOME PROLIFERATOR-ACTIVATED RECEPTORS (PPARs)

Peroxisome proliferator-activated receptors (PPARs) are members of the steroid hormone nuclear receptor superfamily. So far, there are three isoforms that have been identified and cloned, including PPAR-*α*, PPAR-*β*/*δ*, and PPAR-*γ*, and they exhibit different tissue distribution as well as different ligand specificities. PPAR-*α* is mainly expressed in hepatocytes, cardiac myocytes, and proximal tubular epithelial cells of the kidney. PPAR-*γ* expression occurs in adipose tissues and colonic mucosal epithelium. PPAR-*δ* is highly expressed in the placenta and large intestine. They can be activated by polyunsaturated fatty acids [1–4].

PPARs are ligand-activated nuclear receptors and they have been extensively studied in the regulation ofgenes involved in glucose and lipid metabolism. They have been thought to play an important role in the regulation of lipid metabolism, energy balance, inflammation, artherosclerosis and glucose control. Like other nuclear receptor family members, all three members of the PPAR family have distinct functional domains responsible for ligand binding, DNA binding, and coactivator/corepressor binding. They bind to direct repeat 1 (DR1) elements or peroxisome proliferators response elements (PPREs) in the promoter region of target genes and drive the transcription of these target genes [1].

Recent studies have shown that PPARs, including PPAR-*α* and *γ*, play a very important role in the regulation of inflammatory responses, through mechanisms involving transactivation or transrepression of gene expression through activation of transcription factors, including NF-*κ*B, AP1, and NFAT. In this review, we will focus on the regulation of PPAR-*α* on immune responses and their agonists as a potential treatment for autoimmune demyelinating diseases such as multiple sclerosis.

Approximately 350 000 people in the United States have physician-diagnosed multiple sclerosis (MS) [5]. It is the leading cause of neurologic disability in the United States in young adults after trauma, thus most patients suffer from the effects of MS for most of their adult life. The cause of MS remains unknown. An autoimmune process for MS is hypothesized because it shares characteristics of inflammation and demyelination with its animal model, EAE. Epidemiologic studies and studies examining the disease in identical twins also suggest that both environment and genetics influence expression of the disease and play a role in disease pathogenesis [6]. There are now six drugs approved for use in the treatment of MS by the FDA, however none of these agents are a cure for the disease, so the need for better treatment strategies for MS remains [7–10]. In addition, the unfortunate expression of progressive multifocal leukoencephalopathy (PML) in MS patients treated with nataluzimab highlights the need for medications with a proven safety record [11–13].

Several animal models have been used to study MS. In some of these models, disease is induced by viruses, such as Theiler's virus or Borna disease virus [14]. Of the EAE models, the most commonly studied are those established in the Lewis rat and in several susceptible mouse strains. Murine EAE results in a relapsing-remitting disease, similar to the early phase of disease for most MS patients, whereas EAE in the Lewis rat is a monophasic illness. In chronic murine EAE, the pathology observed in the white matter shows much more demyelination than the Lewis rat model, again being more reminiscent of the pathology seen in the CNS of patients with MS. With the advent of transgenic and homologous recombination technology, it is increasingly clear that many powerful molecular tools are becoming available to study the immune response in pathologic processes such as EAE.

## 2. REGULATION OF IMMUNE RESPONSES BY PPAR-*α*

### 2.1. PPAR-*α* expression in immune cells

PPAR-*α* is predominantly expressed in tissues exhibiting high catabolic rates of fatty acids (liver, heart, kidney, and muscle). However, recent studies have shown that it is also expressed in immune cells.

**Monocytes and macrophages**

Chinetti et al. [15] showed that PPAR-*α* is expressed in undifferentiated monocytes and in differentiated human monocyte-derived macrophages. PPAR-*α* is constitutively expressed in the cytoplasm, whereas PPAR-*γ* is predominantly localized in the nucleus. They both were shown to be transcriptionally active after ligand binding to their receptors. Both PPAR-*α* and PPAR-*γ* ligands induce apoptosis of macrophages following activation with tumor necrosis factor-*α*/interferon-*γ*.

**T and B lymphocytes**

PPAR-*α* has been reported to be expressed in T and B lymphocytes [16, 17]. Jones et al. [16] demonstrated that T and B lymphocytes constitutively express PPAR-*α* and PPAR-*γ*. PPAR-*α* is the predominant isoform expressed in lymphocytes, whereas PPAR-*γ* dominates in all cell types of the myeloid lineage. However, PPAR-*α* and PPAR-*γ* are differentially expressed following lymphocyte activation. PPAR-*α* expression was downregulated following T-cell activation while PPAR-*γ* expression increased under the same activating conditions. Exposure to specific ligand determined that PPAR-*α* in lymphocytes effectively transactivates a peroxisome proliferator response element (PPRE) reporter construct. Ligand activation of lymphocyte PPAR-*α* antagonized NF-*κ*B. These observations suggested that a functional PPAR-*α* exists within T cells and B lymphocytes.

**Langerhans cells**

Epidermal Langerhans cells (LCs) play a pivotal role in initiating and maintaining primary immune responses in the skin. Dubrac et al. [18] showed that PPAR-*α* is expressed in immature LC and downregulated in mature LC. Pharmacologic PPAR-*α* activation inhibits LC maturation, migratory capacity, cytokine expression, and the ability to drive T-cell proliferation. Moreover, PPAR-*α* activation inhibits NF-*κ*B but not stress-activated protein kinase/JNK, p38MAPK, and ERK1/2. This study suggested that PPAR-*α* activation by endogenous ligands may provide a molecular signal that allows LC to remain in an immature state.

### 2.2. PPAR-*α* regulation of inflammation and cytokine production

The study of PPAR-*α* deficient mice revealed the relationship between PPAR-*α* and inflammation. Devchand et al. [19] demonstrated that lack of PPAR-*α* activity increases inflammatory responses. They showed that inflammation due to inflammatory agents, including arachidonic acid and LTB4, is prolonged in PPAR-*α* deficient mice as compared to wild-type mice. The *β* and *γ* PPAR subtypes did not compensate for a lack of PPAR-*α* in an LTB4-mediated inflammatory response.

Delerive et al. [20] showed another possible mechanism of PPAR-*α* regulating inflammation. They demonstrated that PPAR-*α* negatively regulates the vascular inflammatory gene response by negative cross-talk with the transcription factors NF-*κ*B and AP-1. They showed that aortic explants isolated from PPAR-*α*-null mice display an exacerbated response to inflammatory stimuli, such as lipopolysaccharide (LPS), as demonstrated by increased IL-6 secretion.

Cytokines are one of the major factors directing T-cell differentiation and play an important role in the pathogenesis of autoimmune diseases. Recent studies have shown that PPAR-*α* regulates the expression of cytokines which are critical in autoimmune disease (see below). Splenocytes harvested from PPAR-*α* agonist, WY14,643, fed and pMOG(35–55) immunized mice showed impaired production of IFN-*γ*, IL-6, and TNF-*α* despite similar proliferative responses, following in vitro restimulation with pMOG(35–55). It was also observed that IL-4 expression in cultures of mitogen-activated splenocytes was increased [21].

Lee et al. [22] reported that the PPAR-*α* agonist, Fenofibrate, repressed IL-17 and interferon-gamma expression and improved colitis in IL-10-deficient mice. PPAR-*α* was found to be expressed in lymphocytes, macrophages, and crypt and surface epithelial cells of the colon. Colonic expression of interferon-gamma and IL-17 genes was decreased in IL-10 deficient mice, when the mice were treated with fenofibrate. Fenofibrate also repressed interferon-gamma and IL-17 expression in isolated T cells, the expression of the genes encoding the chemokines, CXCL10, CCL2, and CCL20, and repressed CXCL10 gene promoter activity in tumor necrosis factor-alpha-treated HT-29 cells.

Jones et al. [23] reported that unliganded PPAR-*α* suppressed T-bet expression and decreased IFN-*γ* production in T cells. They demonstrated that activated CD4(+) T cells lacking PPAR-*α* produce increased levels of IFN-*γ*, but significantly lower levels of IL-2 when compared with activated wild-type CD4(+) T cells.

Another study by Dasgupta et al. [24] suggested that PPAR-*α* increased the activity of GATA-3 and inhibited expression of T-bet, which would be in agreement with prior studies which showed that PPAR-*α* agonists increased IL-4 production by T cells. Interestingly, this study also suggested that the PPAR-*α* agonist gemfibrozil could inhibit clinical signs of EAE in mice deficient in PPAR-*α*, with concomitant upregulation of IL-4 and inhibition of IFN-*γ* [24]. This study did not indicate whether the same changes in T-bet and GATA-3 expression also occurred in PPAR-*α* deficient mice.

Delerive et al. [20] showed fibrate treatment represses IL-6 mRNA levels in LPS-stimulated aortas of PPAR-*α* wild-type, but not of PPAR-*α*-null mice, demonstrating a role for PPAR-*α* in this fibrate action. In human aortic smooth muscle cells, fibrates inhibit IL-1-induced IL-6 gene expression.

### 2.3. Possible mechanisms

Like other transcription factors, PPARs are able to positively regulate gene expression by binding to PPRE as a heterodimer with the retinoic acid X receptor (RXR). In the unliganded state, PPARs are associated with a nuclearreceptor corepressor. In addition, heat shock protein-90 and the hepatitis virus B X-associated protein 2 have been shown to associate with PPAR-*α* and negatively regulate subsequent gene activation [25, 26]. Upon activation, the PPARs undergo a conformational change that results in the dissociation from the corepressor, enabling the PPAR to bind nuclear receptor coactivators. These coactivators then act to reorganize the chromatin templates allowing the basal transcription machinery to gain access to the promoter regions driving transcription of target genes.

**Figure 1:**
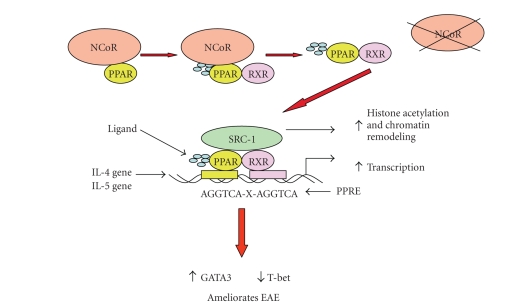
*A model for PPAR-*α*-mediated protection in EAE*. In the presence of PPAR-*α* agonists, PPAR-*α* heterodimerizes with RXR, dissociates from its nuclear corepressor complex, associates with a coactivator complex, and binds to PPREs in the promoter region of IL-4 and/or IL-5. The transactivation of IL-4/IL-5 leads to increased expression of GATA-3 which in turn results in decreased T-bet expression and downregulation of the Th1/Th17 inflammatory response. This shift in the immune response to a Th2-like phenotype results in amelioration of EAE.

In our lab, we have investigated the mechanism by which the PPAR-*α* agonist gemfibrozil induces immune deviation and protects mice from EAE. Similar to the studies by Dasgupta [24], we demonstrated that treatment with gemfibrozil increases GATA-3 and decreases T-bet expression in vitro and directly ex-vivo. These changes correlated with an increase in nuclear PPAR-*α* expression. Moreover, the protective effects of gemfibrozil in EAE were shown to be partially dependent on IL-4 and to occur in a receptor-dependent manner. PPAR-*α* was shown to regulate the IL-4 and IL-5 genes and bound the IL-4 promoter in the presence of steroid receptor coactivator-1 (SRC-1), suggesting transactivation of the IL-4 gene (Figure 1) [27].

PPARs cannot only induce but also repress gene transcription. One recent study showed a sumoylation-dependent pathway mediating transrepression of inflammatory response genes by PPAR-*γ* in macrophages [28]. The initial step of this pathway involves ligand-dependent sumoylation of the PPAR-*γ* ligand-binding domain, which targets PPAR-*γ* to nuclear receptor corepressor (NCoR)-histone deacetylase-3 (HDAC3) complexes on inflammatory gene promoters. This in turn prevents recruitment of the ubiquitylation/19S proteasome machinery that normally mediates the signal-dependent removal of corepressor complexes required for gene activation. As a result, NCoR complexes are not cleared from the promoter and target genes are maintained in a repressed state. This mechanism provides an explanation for how an agonist-bound nuclear receptor can be converted from an activator of transcription to a promoter-specific repressor of NF-*κ*B target genes. However, so far there is no evidence showing that PPAR-*α* is able to repress target genes by this sumoylation-dependent pathway.

Activated PPAR-*α* has been demonstrated to exert anti-inflammatory activities through its ability to antagonize other signaling pathways, in part through the interaction with other transcription factors, including NF-*κ*B, AP-1, and STATs (see below).

Spencer et al. [29] have demonstrated that therapeutic treatment of aged mice with PPAR-*α* activating agents corrected abnormal nuclear NF-*κ*B activity, redu ced lipid peroxide levels, and eliminated the dysregulated expression of cytokines and other genes under NF-*κ*B control.

Delerive et al. showed activation of PPAR-*α* represses both c-Jun- and p65-induced transcription of the human IL-6 promoter. Glutathione S-transferase (GST) pull-down experiments demonstrated that PPAR-*α* physically interacts with c-Jun, p65, and CBP [20]. They further showed that fibrates, synthetic PPAR-*α* activators, induced the expression of the inhibitory protein I*κ*B*α* in human aortic smooth muscle cells as well as in primary human hepatocytes. They demonstrated that fibrates induced I*κ*B*α* in liver in vivo and that this action required PPAR-*α*. Furthermore, fibrate treatment induced I*κ*B*α* protein expression in the cytoplasm and also enhanced IL-1*β*-induced accumulation of I*κ*B*α* protein in the nucleus [30]. These actions of fibrates on I*κ*B*α* expression were accompanied by a decrease in NF-*κ*B DNA binding activity. They further demonstrated that induction of I*κ*B*α* gene transcription by PPAR-*α* is DNA binding-independent. They demonstrated that PPAR-*α* potentiates p65-stimulated I*κ*B*α* transcription in a ligand-dependent manner. PPAR-*α* activation of I*κ*B*α* transcription requires the NF-*κ*B and Sp1 sites within the I*κ*B*α* promoter. PPAR-*α* activation enhances the occupancy of the NF-*κ*B response element in I*κ*B*α* promoter in vivo. VDR-interacting protein 205 (DRIP205) is required to regulate I*κ*B*α* promoter activity [31].

PPAR-*α* was also found to negatively regulate the transcription of T-bet. T-bet is a key regulator of the IFN*γ* gene in Th1 cells. The induction of T-bet expression in CD4(+) T cells was determined to be positively influenced by p38 mitogen-activated protein (MAP) kinase activation, and the presence of unliganded PPAR-*α* effectively suppressed the phosphorylation of p38 MAP kinase. The activation of PPAR-*α* with highly specific ligands relaxed its capacity to suppress p38 MAP kinase phosphorylation and promoted T-bet expression [23]. This observation conflicts with the observation of Dasgupta [24] and our own work.

Lee et al. found that four PPAR-*α* activators suppressed lipopolysaccharide-stimulated STAT1 phosphorylation and nuclear factor binding to *γ*-interferon-activated sequence/interferon-*α*-stimulated response element sites known to contain STAT binding sites. PPAR-*α* activators also suppressed lipopolysaccharide-stimulated tumor necrosis factor-*α* and monocyte chemoattractant protein-1 transcription and release [32].

In addition to PPAR-*α* dependent transcriptional regulation, Selim et al. [33] showed that fibrates upregulate TRB3 in lymphocytes independent of PPAR-*α* by augmenting CCAAT/enhancer-binding protein *β* (C/EBP-*β*) expression. They demonstrated that fibrates upregulate TRB3 expression (a protein that interferes with insulin-induced activation of AKT), in mitogen-activated lymphocytes of both wild type and knockout mice, suggesting that upregulation of this protein occurs in a PPAR-*α*-independent manner.

Dasgupta et al. [24] showed gemfibrozil inhibited the encephalitogenicity of MBP-primed T cells and switched the immune response from a Th1 to a Th2 profile independent of PPAR-*α*. Gemfibrozil consistently inhibited the expression and DNA-binding activity of T-bet, and stimulated the expression and DNA-binding activity of GATA-3, a key regulator of IL-4. Gemfibrozil treatment decreased the number of T-bet-positive T cells and increased the number of GATA-3-positive T cells in the spleens of donor mice. Gemfibrozil was shown to have an inhibitory effect on the invasion of T-bet-positive T cells into the spinal cord of EAE mice. Furthermore, they demonstrate that the differential effect of gemfibrozil on the expression of T-bet and GATA-3 was due to its inhibitory effect on NO production.

## 3. PPAR ALPHA AND AUTOIMMUNE ENCEPHALOMYELITIS

Organ-specific autoimmune diseases, such as multiple sclerosis (MS) and its animal model, are mediated by IFN-*γ* and/or IL-17 producing CD4 T helper cells. Since PPAR-*α* regulates inflammation and cytokine production, PPAR-*α* agonists have been tested as a potential treatment for autoimmune diseases.

Lovett-Racke et al. [34] demonstrated that PPAR-*α* agonists can be used as a therapy for autoimmune disease. They demonstrated that PPAR-*α* agonists can increase the production of the Th2 cytokine, IL-4, and suppress proliferation by TCR transgenic T cells specific for the myelin basic protein Ac1-11, as well as reduce NO production by microglia. Oral administration of gemfibrozil and fenofibrate inhibited clinical signs of experimental autoimmune encephalomyelitis. More importantly, gemfibrozil was shown to shift the cytokine secretion of human T-cell lines by inhibiting IFN-*γ* and promoting IL-4 secretion. These results suggest that PPAR-*α* agonists, such as gemfibrozil and fenofibrate, may be attractive candidates for use in human inflammatory conditions such as multiple sclerosis.

In another study, Dasgupta et al. [24] demonstrated that gemfibrozil ameliorates relapsing-remitting EAE independent of PPAR-*α*. They showed that clinical signs of EAE, infiltration of mononuclear cells, and demyelination were significantly lower in mice receiving gemfibrozil, suggesting gemfibrozil may find therapeutic use in multiple sclerosis.

Interestingly, PPAR-*α* expression in T cells was suggested to mediate gender differences in development of T-cell-mediated autoimmunity. Dunn et al. [35] showed that PPAR-*α* is more abundant in male as compared with female CD4(+) cells and that its expression is sensitive to androgen levels. Upon induction of EAE, male PPAR-*α* (−/−) mice developed more severe clinical signs than females. These results suggest that males are less prone to develop Th1-mediated autoimmunity because they have higher T-cell expression of PPAR-*α*.

Xu et al. [36] investigated the effects of PPAR-*α* agonists on primary mouse microglia, a cell type implicated in the pathology of MS and EAE. They demonstrated that PPAR-*α* agonists inhibited the secretion of IL-1*β*, TNF-*α*, IL-6, and IL-12 p40 and the chemokine MCP-1 by LPS-stimulated microglia. Retinoid X receptors (RXRs) physically interact with PPAR-*α* receptors, and the resulting heterodimers regulate the expression of PPAR-responsive genes. They demonstrated that the PPAR-*α* agonists ciprofibrate, fenofibrate, gemfibrozil, and WY14,643 inhibited NO production by stimulated microglia in a dose-dependent manner. Furthermore, a combination of 9-cis RA and the PPAR-*α* agonist fenofibrate cooperatively inhibited NO production by these cells. This study suggested that PPAR-*α* and RXR agonists might have benefit as a therapy in MS, where activated microglia are believed to contribute to disease pathology.

Other than microglia cells, they also investigated the effects of PPAR-*α* agonists on primary mouse astrocytes [37]. They observed similar inhibition on cytokine production by PPAR-*α* agonists. PPAR-*α* agonists inhibited the secretion of TNF-*α*, IL-1*β*, and IL-6 by LPS-stimulated astrocytes. Additionally, fenofibrate inhibited NF-*κ*B DNA binding activity, suggesting a mechanism by which PPAR-*α* agonists may regulate the expression of genes encoding these proinflammatory molecules. Retinoid X receptors (RXRs) physically interact with PPAR-*α* receptors, and the resulting heterodimers regulate the expression of PPAR-responsive genes.

They further demonstrated that fenofibrate suppression of EAE was associated with decreased expression of IL-12 family cytokine mRNAs as well as mRNAs encoding TLR4, CD14, and MyD88. They showed that the PPAR-*α* agonist fenofibrate inhibited the secretion of IL-12p40, IL-12p70 (p35/p40), IL-23 (p19/p40), and IL-27p28 by lipopolysaccharide-stimulated microglia. Furthermore, fenofibrate inhibited microglial expression of CD14 which plays a critical role in TLR signaling [38].

## 4. CONCLUSION

The functional expression of PPAR-*α* by several immune cell types suggests that this receptor may play a very important role in regulation of immune responses. Recent studies demonstrate that PPAR-*α* regulates different aspects of immune responses, including inflammation and cytokine production. Moreover, several studies showed evidence that PPAR-*α* agonists have potent effects in regulating immune responses and ameliorating EAE. However, the detailed mechanisms have not been completely delineated. Better understanding of the molecular mechanism by which PPAR-*α* regulates cytokine pathways in immune cells will be very helpful for further development of PPAR-*α* agonists as a therapy for autoimmune diseases.
